# Avoidable pitfalls on the path to health financing self-reliance in low-income and middle-income countries

**DOI:** 10.1136/bmjgh-2025-021270

**Published:** 2025-11-29

**Authors:** Edwine Barasa, Jane Chuma, Justice Nonvignon, Olusoji O Adeyi

**Affiliations:** 1 Health Economics Research Unit, KEMRI-Wellcome Trust Research Programme, Kilifi, Kilifi County, Kenya; 2 Center for Tropical Medicine and Global Health, Nuffield Department of Medicine, University of Oxford, Oxford, England, UK; 3 Health and Nutrition, World Bank, Zambia Country Office, Lusaka, Lusaka, Zambia; 4 University of Ghana, Accra, Ghana; 5 Management Sciences for Health, Arlington, Texas, USA; 6 Resilient Health Systems, Washington, District of Columbia, USA; 7 Department of International Health, Johns Hopkins Bloomberg School of Public Health, Baltimore, Maryland, USA

**Keywords:** Global Health, Health policy, Health systems

## Abstract

Low-income and middle-income countries (LMICs) are facing an urgent and complex challenge: how to transition to greater self-sustainability in health financing amid declining donor support. While this shift is inevitable, the policy responses it elicits carry significant implications for health system equity and access. This commentary highlights four policy choices increasingly observed in LMICs that we argue are unacceptable in the pursuit of sustainability. These include: (1) shifting the financial burden to out-of-pocket payments; (2) over-reliance on contributory health insurance schemes; (3) displacement of basic primary healthcare services; and (4) abandoning community-based service delivery in favour of facility-centric models, undermining the integrity of people-centred health systems. We argue that while short-term fiscal pressures may push countries towards these decisions, they ultimately erode health gains, exacerbate inequities and threaten progress towards universal health coverage. We call on policymakers to adopt evidence-informed approaches that enhance efficiency, protect the most vulnerable, prioritise public financing and preserve the core values of inclusive and equitable health systems during this critical transition.

**SUMMARY BOX**

- Countries are considering policy responses to donor cuts that include regressive financing and service delivery shifts with harmful equity implications.
- Countries should avoid shifting financial burdens to households through user fees and recognise the limitations of contributory insurance in low-income and middle-income countries and secure tax-based funding for critical services.
- Countries should ensure integration of services into primary healthcare preserves essential basic care and does not crowd out core interventions.
- Countries should formalise and invest in community health systems as a sustainable, equitable service delivery strategy.

## Introduction

Low-income and middle-income countries (LMICs) are confronting a new fiscal reality in the wake of declining external assistance for health. Recent reductions in donor funding, most notably from the United States Government (USG) and other major global health funders, have created what has been referred to as funding cliffs.^1^ These sudden and significant drops in aid threaten the delivery of critical health services, risking the reversal of decades of progress in the fight against HIV, tuberculosis, malaria and in reproductive, maternal, newborn, child and adolescent health (RMNCAH).^2^,^4^

The trajectory is clear: a return to previous levels of external funding is unlikely, especially because declines in external funding for health were already in progress before the sudden cuts experienced at the beginning of 2025.^5^ LMICs must now take deliberate steps to transition towards greater self-sustainability in health financing. This transition calls for immediate, medium-term and long-term measures, ranging from short-term mitigation strategies to absorb and mitigate the impact of funding cuts, to more structural reforms aimed at adapting and transforming health systems towards self-reliance and sustainability through mobilising domestic resources for health, improving efficiency in the allocation and use of available funds, and redesigning service delivery models.

As countries chart this new course, they will face a range of complex choices. However, choices that compromise widely recognised goals of health systems—equity, access, efficiency, quality and financial protection—must be considered unacceptable. While these goals may be contested, and their relative importance depends on the values and power dynamics of political and social actors, they nonetheless represent shared aspirations articulated in global and national policy frameworks. In the pursuit of self-sustainability, there is a real risk that some decisions may undermine fundamental health system goals, particularly access, equity, efficiency and financial risk protection.

In this paper, we draw from our collective reflections, based on interactions and engagements with several LMIC governments and policymakers on this issue, to highlight four shifts that, from an equity-focused public health perspective, we consider unacceptable and avoidable on the path to self-sustainability. These policy pitfalls, while potentially expedient in the short term, are ultimately counterproductive and risk entrenching and exacerbating inequities in health systems. We urge policymakers to exercise caution and foresight, ensuring that the drive for financial sustainability does not come at the cost of the very populations that health systems are meant to serve. We acknowledge, however, that perceptions of what is acceptable may vary depending on the priorities and values of different stakeholders involved in health system decision-making.

## Shifting the financial burden to households

As donor funding declines, countries are under increasing pressure to identify alternative sources of revenue for the health sector. While this fiscal pressure is real, it must not justify a return to one of the most inequitable forms of health financing: out-of-pocket (OOP) payments at the point of care. This is a real concern for countries with limited fiscal space, and those whose health financing structures predominantly rely on OOP payment. For instance, external funding for health accounts for only 7% of current health expenditure in Nigeria but 65% in Malawi. In both countries, external funding has been a major financier of services for HIV/AIDS, tuberculosis and malaria, among others. Furthermore, OOP payments are the predominant source of funding in Nigeria, accounting for 76% of current health expenditure, but only 12% in Malawi.^6^ This implies that there is a real risk that as donor funding declines, countries shift the financial burden directly to households. Reaching the goals of financial self-reliance in such different contexts requires country-specific calibration of the populations’ expectations and deployment of domestic resources while protecting the most vulnerable.

Introducing user charges may appear to be a pragmatic solution, especially for health facility managers facing immediate funding shortfalls, as it can seem to be the only option to maintain service delivery. However, reliance on user fees is ultimately a regressive and inequitable approach. It places a disproportionate burden on the poor and undermines health system goals of equity and financial protection. Modelling estimates indicate that the USG aid cuts could push 5.7 million Africans into poverty; this could increase to 19 million Africans by 2030.^2^ OOP payments create significant financial barriers to access, particularly for the poorest and most vulnerable populations. For many households, even modest fees can deter the use of essential services or lead to catastrophic health expenditures that push families into poverty. More critically, reliance on OOP payments effectively prioritises the needs of those who can afford care over those who cannot. Given that the withdrawn donor funds have been funding access to life-saving interventions, such as antiretroviral therapy for HIV, treatment for tuberculosis and malaria prevention, shifting to user fees risks rolling back the hard-won gains in equitable coverage with these interventions and will compromise population health outcomes.

This shift is not only ethically indefensible, but also inefficient. There is overwhelming evidence that user fees mobilise very limited resources for the public health sector, making it an inefficient mechanism for revenue mobilisation.^7^ Therefore, while countries must explore strategies to raise additional domestic resources, reinstating or increasing user fees is an unacceptable trade-off on the path to self-sustainability. Policymakers must resist the temptation to shift the financial burden onto patients and instead prioritise progressive, pooled and prepaid financing mechanisms (eg, tax funding) that uphold equity and protect the poor. While, where feasible, countries should explore opportunities to mobilise additional domestic resources, this should be accompanied by increased prioritisation of health within existing government budgets. It is equally important that countries improve the efficiency of spending available resources and there are opportunities to do so. For instance, there is evidence that public finance management reforms aimed at improving the efficiency of health system financing can be implemented with positive impacts in resource-constrained settings.^8^

### Shift to contributory health insurance

In response to dwindling donor funding, many countries are considering expanding contributory health insurance schemes, whether public social health insurance or private insurance, as a means to sustain previously donor-funded health services.^9 10^ The appeal of this option lies in its promise of financial protection through prepayment and risk pooling. There is also the false belief that this approach alone is the surest way to raise additional resources and expand fiscal space for health. However, for most LMICs, this approach risks creating a false sense of coverage and deepening inequities if the funding of these services is expected to be largely funded by enrolled member premium contributions. Moreover, the revenue-generating potential of contributory schemes in LMICs is extremely limited. On average, such schemes contribute about 5% of total health expenditure, making them an inefficient resource mobilisation mechanism and inadequate replacement for donor funds.^6^

The structural limitations of contributory health insurance in LMICs are well documented.^11 12^ High levels of poverty and informality in the labour market mean that large segments of the population are unable to contribute to insurance schemes, resulting in chronically low enrolment rates.^11 12^ Those who do enrol are disproportionately drawn from formal sector workers and higher-income groups, leaving behind the poor and most vulnerable.^13^ For instance, mean population enrolment in a health insurance scheme is low (8%) in Africa, characterised by substantial inequalities, with the richest quintile having a fourfold level of population enrolment, compared with the lowest quintile.^13^ Further, these schemes are often de facto voluntary, which introduces adverse selection, increases administrative inefficiencies and threatens financial sustainability.^11 12^

Attempting to shift the burden of funding previously donor-funded services, such as HIV care and management, and TB treatment, onto contributory schemes would exacerbate their fragility and fail to protect the population. While these services may appear covered on paper (de jure), the reality (de facto) is that they will be underfunded or entirely inaccessible to many.

This shift risks marginalising the uninsured majority and institutionalising a two-tier system of care, where the well-off access these critical services, while the poor are left with fragmented or no coverage at all. It is therefore imperative that countries recognise the limitations of contributory health insurance and provide for tax funding of these services within these insurance schemes, should they choose to integrate these services within existing health insurance schemes. Sustainable health financing must be anchored in non-contributory, tax-funded mechanisms that ensure access based on need, rather than ability to pay.

### Displacement of basic primary healthcare (PHC) services

In the face of declining donor funding, many countries are rightly seeking to integrate and reprioritise previously donor-supported programmes, such as HIV, TB and malaria, into their broader PHC platforms.^14^ The verticalised delivery of these services has meant that core health system functions—governance, financing, human resources, commodities and supply chain, information systems and service delivery for these services have been fragmented and inefficient. Integration efforts will need to address fragmentation across these functions. While integration holds the potential for greater efficiency and continuity of care, if poorly planned or implemented, it carries a serious risk: the crowding out of essential, basic PHC services.

The PHC system is the cornerstone of equitable, accessible and cost-effective healthcare delivery.^15^ It provides the first point of contact for communities and addresses a wide range of preventive, promotive and curative health needs. However, without deliberate planning, the absorption of previously donor-funded services into this platform can distort priorities and divert already constrained resources away from foundational PHC services such as immunisation, antenatal care, child health and treatment of common illnesses. This not only compromises access, but also compromises equity and efficiency, since PHC has been shown to be among the most equitable and efficient healthcare delivery platforms.^15^

The risk of displacement is particularly high when integration is not guided by evidence-informed prioritisation. Faced with limited fiscal space, countries will have to make difficult decisions about which services to retain, scale back or discontinue. These decisions must be grounded in robust data on cost-effectiveness, equity implications, and health system impact to ensure that available resources are directed toward services that yield the greatest health benefit. Integration should not become a zero-sum game, where the expansion of one set of services leads to the erosion of another. Rather, countries must adopt a deliberate, phased and evidence-informed approach to integration, one that safeguards essential PHC services while expanding access to previously donor-funded programmes.

### Abandoning community-based approaches for facility-centred models

The success of many donor-supported health programmes, particularly in HIV, TB, malaria and RMNCAH, has been underpinned by strong community-based delivery models.^16 17^ Community health workers (CHWs) play critical roles in TB service delivery such as active TB case finding, sample collection and transport and treatment support,^18^ and in HIV care—referring and linking patients to care and providing psychosocial support.^16 17^ These same CHWs also play a critical role in the delivery of essential PHC services using people-centred approaches.^19^ These approaches, anchored in community health systems and delivered through CHWs, have proven instrumental in expanding access to services, especially among remote and marginalised populations.^16^,^18^ They have enabled early detection, improved adherence, reduced disease burden and strengthened health promotion at the grassroots level.^16^,^18^

However, in many LMICs, community health systems remain structurally disconnected from the formal health sector.^20^ CHWs are often not formally recognised as part of the health workforce; they lack standardised training, remuneration and career progression pathways.^20^ Their work is typically supported by external funding, with limited government ownership or integration into national health workforce strategies.^21^

As donor funding for community-based programmes recedes, there is a real risk that governments will retreat from these people-centred approaches and revert to a predominantly facility-based model of care. This would be a costly mistake. A shift away from community-based care towards exclusively facility-centric delivery risks rolling back hard-won gains in equitable service access. It would disproportionately affect populations who face geographic, financial and social barriers to accessing care at health facilities, further entrenching inequities. Further, a shift from a community centric to a facility-centric model will compromise the efficiency of health systems given that community health systems are more cost-effective compared with facility-based approaches in the delivery of basic PHC services.^22^ Moreover, facility-based systems alone are insufficient to achieve universal health coverage goals, especially in rural or underserved areas where proximity to care remains a critical barrier. Countries should instead strive to have optimal service delivery systems that integrate and extend facility-based care with community-based approaches. The optimal balance between facilities and community outreach will vary with context, and decentralised or local health system levels should play an increasing role in shaping appropriate delivery models.

Rather than disinvesting in community-based delivery, countries should seize this moment to formalise and institutionalise community health systems as a core part of their national health infrastructure, as this is likely a more affordable and sustainable path, if well implemented, compared with facility-based approaches. This includes recognising CHWs as a standard cadre, integrating them into health workforce planning and financing their roles through domestic resources. This is not to say, however, that countries should inherit existing donor-funded community health system arrangements without adaptation. Countries should interrogate these systems and evolve them to enhance efficiencies and alignment with broader health systems.

## Conclusion

The transition towards greater domestic self-sufficiency in health financing is both necessary and urgent for LMICs. However, the path to sustainability must not be littered with choices that compromise the foundational goals of health systems—equity, access, quality, efficiency and financial protection. The shifts outlined in this commentary—reverting to OOP payments, over-reliance on contributory insurance, displacement of basic PHC services and the abandonment of community-based approaches—represent false economies. They may appear fiscally expedient in the short term, but they are ultimately self-defeating, inefficient, entrenching inequities and eroding gains made in improving population access and coverage with critical health interventions.

A sustainable transition must be guided by the principles of progressive universalism: protecting the most vulnerable, expanding coverage through publicly financed prepayment mechanisms and preserving the gains made through decades of global health investment. This requires deliberate policy choices, evidence-informed prioritisation and a political commitment to inclusive, equitable health systems. As development assistance declines, governments must resist the allure of short-term fixes that undermine long-term progress. Instead, they should seize this moment as an opportunity to reimagine and reconfigure health systems and financing models in ways that ensure that no one is left behind.

## Data Availability

There are no data in this work.
